# Hippocampus-dependent place learning enables spatial flexibility in C57BL6/N mice

**DOI:** 10.3389/fnbeh.2012.00087

**Published:** 2012-12-27

**Authors:** Karl R. Kleinknecht, Benedikt T. Bedenk, Sebastian F. Kaltwasser, Barbara Grünecker, Yi-Chun Yen, Michael Czisch, Carsten T. Wotjak

**Affiliations:** Max Planck Institute of PsychiatryMunich, Germany

**Keywords:** spatial memory, cognitive flexibility, response learning, place learning, hippocampus, MRI volumetry, ibotenic acid

## Abstract

Spatial navigation is a fundamental capability necessary in everyday life to locate food, social partners, and shelter. It results from two very different strategies: (1) place learning which enables for flexible way finding and (2) response learning that leads to a more rigid “route following.” Despite the importance of knockout techniques that are only available in mice, little is known about mice' flexibility in spatial navigation tasks. Here we demonstrate for C57BL6/N mice in a water-cross maze (WCM) that only place learning enables spatial flexibility and relearning of a platform position, whereas response learning does not. This capability depends on an intact hippocampal formation, since hippocampus lesions by ibotenic acid (IA) disrupted relearning. *In vivo* manganese-enhanced magnetic resonance imaging revealed a volume loss of ≥60% of the hippocampus as a critical threshold for relearning impairments. In particular the changes in the left ventral hippocampus were indicative of relearning deficits. In summary, our findings establish the importance of hippocampus-dependent place learning for spatial flexibility and provide a first systematic analysis on spatial flexibility in mice.

## Introduction

Life often requires navigation in complex environments. Humans and rodents have developed a number of strategies to do so, with great importance of place and response learning (Tolman et al., 1946; Maguire et al., 1998; Hartley et al., 2003; Collett and Graham, 2004; Etchamendy and Bohbot, 2007; Liu et al., 2011). *Place learning* is a hippocampus-dependent navigation strategy, characterized by the use of environmental information incorporated into a cognitive map to locate a destination (O'Keefe et al., 1975; Morris et al., 1982; Eichenbaum et al., 1990; Dupret et al., 2010; Gutierrez-Guzman et al., 2011). It is described to be flexible since it does not rely on the starting position of the subject. *Response learning*, in contrast, depends on the starting position and is therefore a less-flexible strategy. It is based on stimulus-response guided navigation and requires intact basal ganglia (Packard et al., 1989; McDonald and White, 1994; Packard and McGaugh, 1996; Packard, 1999; McDonald and Hong, 2004; Tzavos et al., 2004; Jacobson et al., 2012).

Navigational strategies have to be flexible in order to allow for adaptation to changing environments. Spatial flexibility can be assessed upon reversal learning and strategy switching (Oliveira et al., 1997; Ragozzino et al., 1999; McDonald et al., 2001; Ragozzino, 2007; Ramos, 2010). *Reversal learning* stands for a modification in spatial orientation on the basis of the same navigational strategy as initially employed (e.g., place learning → place learning). In contrast, *strategy switching* is achieved by an alternation in the navigational strategy (e.g., place learning → response learning).

In rodents, systematic analyses of spatial flexibility were primarily done in rats. The enormous number of animal models based on elaborate genetic engineering has raised great interest in performing similar experiments in mice. However, surprisingly little is known about spatial flexibility in mice.

Here we study reversal learning and strategy switching on the basis of place and response protocols in C57BL6/N mice with or without ibotenic acid (IA)-lesion of the HPC. We relate the behavioral deficits to volumetric changes of the HPC measured *in vivo* by means of manganese-enhanced magnetic resonance imaging (MEMRI). To clearly distinguish between the different navigation strategies, we establish and validate a water-cross maze (WCM) task, which is based on the classical Tolman maze (Tolman et al., 1946; Schroeder et al., 2002; Packard and Wingard, 2004; Wingard and Packard, 2008). We demonstrate that, for C57BL6/N mice, relearning of a platform position is only possible on the basis of place learning, but not response learning, irrespective of the originally learned strategy, and that an intact HPC is essential for this spatial flexibility.

## Materials and methods

### Animals

A total of 122 male C57BL6/N mice (Charles River, Germany), 6–7-weeks-old, were single housed in standard macrolon cages (type II) with food and water available *ad libitum*. The mice were maintained on a reversed 12 h light/dark cycle (lights off at 09:00) in a temperature- and humidity-controlled room. After arrival, the animals were allowed to become accustomed to the local animal facility and the reversed light/dark cycle for a period of at least 10 days. All behavioral training occurred during the dark phase of the circadian cycle, corresponding to the activity phase. Mice were kept in a separate room adjacent to the test room.

### Water-cross maze

**Figure 1:**
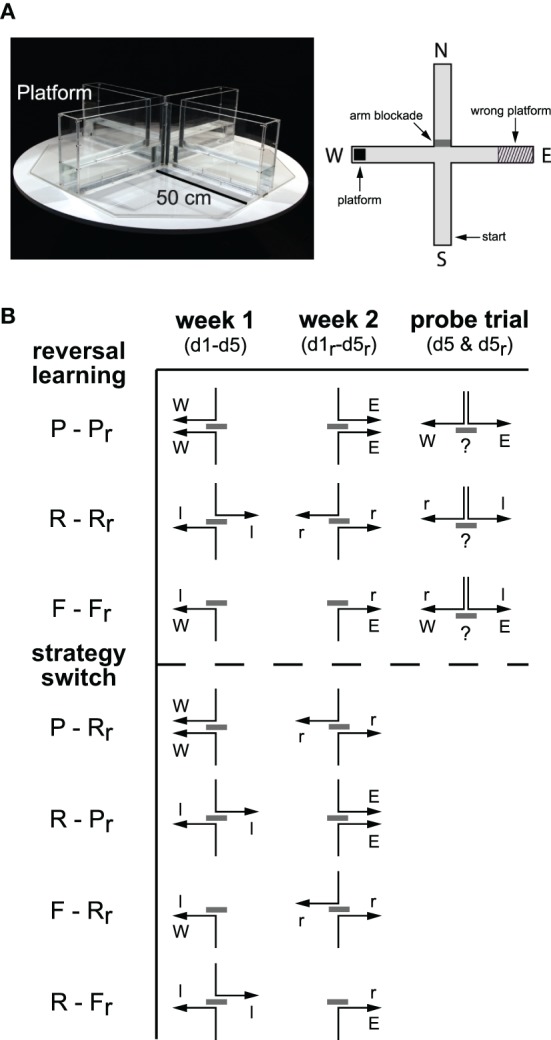
**Spatial navigation in the water-cross maze. (A)** Photo and schematic drawing of the water-cross maze (WCM) depicting an exemplary constellation of starting point, blocked arm, platform position, and wrong platform area oriented toward the four cardinal points [North (N), East (E), South (S), West (W)]. Note that a flexible wall was used for arm blockade of the arm opposite to the start arm, thus rendering the cross-maze a T-maze during each trial. **(B)** Scheme of the track to the platform for 5 days learning (d1–d5) in the 1st week, 5 days relearning (d1_r_–d5_r_) in the 2nd week and a probe trial on day 5 after the learning trials in both weeks (d5 and d5_r_). Place (P), response (R), and free choice (F) learning protocols were employed during learning and relearning (r) within one modality (reversal learning) or between modalities (strategy switching). Gray bars represent the arm blockade, arrows represent the reinforced track and cardinal points [West (W), East (E), left (l), right (r)] for allocentric and egocentric strategies, respectively.

The WCM (custom made, MPI of Psychiatry, Germany; Figure 1A) has four arms forming a cross. It is made from 0.5 cm thick clear acrylic glass to allow for visual orientation via distal extra-maze cues in the experimental room. Each arm is 10 cm wide and 50 cm long and enclosed by 30 cm deep side and end walls. The arms are labeled North, East, South, and West in clockwise order. In our setup, a removable clear acrylic glass shield was used to block the entrance into the arm opposing the starting position. Thus the cross-maze becomes a T-maze that forces the mouse to turn right or left rather than swimming straight ahead. An 8 × 8 cm^2^ escape platform made of the same clear acrylic glass was submerged in one end of an arm 1 cm under the water surface invisible to the mice. Each test day, the maze was filled with fresh tap water (23°C) up to a height of 11 cm. A stick with a 9 × 9 cm^2^ metal grid attached was used to remove the animal from the maze. The testing room was dimly lit by four lights in every corner of the room emitting indirect regular spectrum light (14 lux at the level of the mouse). The room contained a sufficient number of distal visual cues e.g., a sink, a small gray cabinet, tubes at the ceiling and the walls in a non-specific arrangement. There were no dominant cues such as light or acoustic gradients.

#### General procedure

Each animal was transported from the holding room into the adjacent test room right before each trial. Mice were gently inserted into the water facing the wall at the end of the start arm and online assessment of the trial was started. The experimenter, wearing a green lab coat, remained motionless ~20 cm behind the current start arm to not be an indicator for the platform position. After the animal climbed onto the platform, it was directly removed with the stick, placed back into its home cage, transferred back to the holding room and placed under an infrared light to dry and warm. Only half of the cage was under infrared light so that the mice could actively avoid overheating. If the animal could not find the platform within 30 s it was guided to it with the same stick used for removal and was taken out of the maze after a 5 s rest on the platform. To avoid olfactory cuing, the water was stirred and the maze's walls were wiped with a soft cloth after each trial. In addition, the water was exchanged between all four arms of the maze every three trials. Animals were tested in cohorts of 6–8, resulting in an inter-trial interval of 10–14 min for an individual animal.

#### Learning protocols

Every animal passed through 30 trials during the learning (week 1, d1–d5) and the relearning (week 2, d1_r_–d5_r_; Figure 1B) with six trials a day over the course of 5 days. In-between learning and relearning, they had 2 days of rest. Animals were trained in one out of three training protocols each week: the place learning (P) protocol, the response learning (R) protocol, and the free choice learning (F) protocol. As schematically depicted in Figure 1B, groups underwent learning in week 1 (d1–d5) and relearning in week 2 (d1_r_–d5_r_) in the same (reversal learning; e.g., P-P_r_) or a different modality (strategy switching; e.g., P-R_r_). Probe trials were conducted after the end of the 5-day training periods as indicated in Figure 1B.

***Place learning protocol.*** This protocol reinforced the usage of information from the surrounding extramaze environment to reach the platform. This room-coordinate-dependent strategy enabled the mice to locate the constant platform position from both starting points (place; Figure 1B, P/P_r_). The starting position varied in a pseudorandom manner between South and North (odd days: N-S-S-N-N-S, even days: S-N-N-S-S-N). During week 1, the platform was located in the West arm, in the 2nd week in the East arm (Figure 1B). An *a priori* side bias can be excluded giving the fact that nearly half of the animals ever tested in our WCM had chosen the West or the East arm, respectively, during the first run on the first testing day (89 animals the West, 93 animals the East, and 30 animals the Start arm).

***Response learning protocol.*** Upon response learning, mice employed a body turn-based strategy to navigate to the platform. The starting position varied in a pseudorandom manner between South and North (odd days: N-S-S-N-N-S, even days: S-N-N-S-S-N). During week 1, the position of the platform was altered in a way that the animal had to perform a body turn (response) to the left in order to reach the platform (Figure 1B, R). During relearning in the 2nd week, the platform was placed in a way that a body turn to the right was required (Figure 1B, R_r_).

***Free choice protocol.*** This protocol allowed for the use of extramaze-cues and body turns to solve a WCM trial. The starting position remained always in the South. In the 1st week, the platform position was constantly in the West (or to be reached by left body turns; Figure 1B, F) whereas it was relocated to the East arm in the 2nd week (or to be reached by right body turns; Figure 1B, F_r_).

***Probe trials.*** Animals started from the North without an escape platform. Behavior was recorded over 30 s for offline analysis. The starting position was only completely new for animals trained with the free choice protocol.

#### Performance parameters

Learning performance was assessed by three basic parameters: accuracy, latency, and number of wrong platform visits. In order to describe the animals' behavior in more detail, three additional variables were deduced from the accuracy, i.e., the number of accurate learners, start bias, and the number of biased starters. For the probe trial, the number of animals that chose one or the other search strategy was counted.

***Accuracy and start bias.*** Every arm entry was scored online by the experimenter. An entry was counted if the whole body excluding the tail was inside one arm. A second entry into the same arm was only counted if the animal had completely left the arm before. A trial was scored as accurate (i.e., value 1), if the animal entered directly the arm containing the platform and climbed onto it. Deviant behavior was counted as non-accurate (i.e., value 0). Accuracy was described as the percentage of accurate trials on each day per animal. An animal reached the criterion of an accurate learner, if it accomplished more than 83% accurate trials per day (i.e., ≥5 out of 6 trials). The number of *accurate learners* was described as the sum of all animals exceeding the threshold on each day. The *start bias* was described as the absolute value of the sum of accurate trials from the South arm minus the sum of accurate trials from the North arm |∑ (accurate North trials) − ∑ (accurate South trials)|. An animal with a daily score ≥2 was considered to be biased. The number of *biased starters* was calculated each day on the basis of the described threshold.

***Latency.*** Latency was described as the arithmetic mean of the time until the platform was reached averaged over the six trials per day. If the animal could not climb on the platform within 30 s, we assumed 31 s for calculation.

***Wrong platform visits.*** Wrong platform visits were counted if the animal entered the outer third of the arm opposite to the platform arm. Another wrong platform visit was only accounted for if the animal left the area before with all four legs. The number of wrong platform visits was summed up.

***Searching strategy.*** For the probe trial, the first entry into an arm from the starting arm was used to classify the animals to a searching strategy. If the animal used the same turn as during training, it was assigned to the response learners, if it swam to the same place, it was assigned to the place learners. The numbers of animals for each strategy were counted.

### Surgery

Three independent cohorts of IA-treated and vehicle-treated (Veh) animals underwent surgery. For anesthesia we injected the mice with a combination of ketamine/xylazine [50 mg/kg ketamine (Belapharm GmbH, Vechta, Germany) +40 mg/kg xylazine (Rompun®, Bayer Vital GmbH, Leverkusen, Germany)]. Anaesthetized animals were mounted to a stereotactic frame (TSE-Systems, Heidelberg, Germany) and HPC lesions were accomplished by bilateral injection of IA with a microinjector pump (UltraMicroPump III + Micro4 Controller, World Precision Instruments Inc., Saratosa, FL, USA). We injected 1.9 μg IA (Sigma Aldrich, Germany) dissolved in Phosphate Buffered Saline (10 μg/μl, pH = 7.4) or pure Phosphate Buffered Saline as control into 3 sites of each HPC going from anterior to posterior and from dorsal to ventral [(1) AP −1.2 mm, L ±1.2 mm, V 2.0 mm, (2) AP −2.5 mm; L ±2.5 mm; V 2.2 mm, (3) AP 3.3 mm, L 3.1 mm, V 4.1 mm]. One injection (0.19 μl) took 2 min followed by 3 min, when the cannula was left in place to allow for diffusion. The wound was disinfected with Braunoderm® (B.Braun, Melsungen, Germany) and closed with sutures. Mice were treated with the analgesic Meloxicam (0.5 mg/kg in 0.2 ml saline, s.c.) and xylocaine (spray) before surgery and by Meloxicam via the drinking water (0.5 mg/kg in 5 ml tap water) during three post-surgery days. Mice were allowed to recover from surgery for 21 days before starting the behavioral experiments (cf. Figure 4A).

### Behavioral screening

All animals that had undergone surgery were screened for general consequences of bilateral lesions of the HPC 3–4 weeks later (Figure 4A). To test for basic locomotion and exploration, animals were exposed to an open field and a holeboard test on two consecutive days. Further we checked anxiety-like behaviors in a light-dark test and hyperarousal in a startle apparatus on the last day. The equipment and the experimental procedures were previously described elsewhere (Kamprath and Wotjak, 2004; Golub et al., 2009; Thoeringer et al., 2010).

#### Open field and holeboard test

Animals were tested in an open field (L26 × W26 × H40 cm, TruScan, Coulbourn Instruments, Allentown, PA, USA) consisting of a white floor and transparent Plexiglas-walls. Two infrared sensor rings 1.5 cm apart from each other measured the movements in the horizontal and vertical direction. For the holeboard testing an elevated floor with 16 holes was put inside the box. The exploration of the holes was monitored with a third sensor ring added to the apparatus. Boxes and beams were surrounded by additional opaque Plexiglas-walls. The beam rings were connected to a computer via an interface and behavior was recorded using TrueScan Software (V.99; Coulbourn Instruments). The open field test was conducted in darkness while during holeboard test the illumination was 15 lux at the level of the mouse.

Mice were placed from their home cages into the center of the box and three-dimensional movements were automatically recorded over the course of 15 min in the open field and 30 min in the holeboard test. After the trial animals were replaced in their home cages and the setup was cleaned thoroughly with soap and water.

The implemented algorithms of the TrueScan software were used for quantification of the following parameters in both tests: Immobility time, distance traveled, number of rearings, and duration of rearings. For the open field test, the time and distance moved in the center was additionally calculated (expressed as the percentage of total time and distance). For the holeboard test, the number and duration of nose pokes was additionally assessed to the above mentioned parameters.

#### Light-dark test

Mice were tested in a box (L46 × W27 × H30 cm) that was divided into two parts: 2/3 of the box was brightly illuminated (700 lux) by cold light lamps (light compartment) while the other 1/3 remained dark (dark compartment). The light compartment consisted of three white opaque walls made of Plexiglas, while all other walls were made of black plastic. A tunnel connected the two compartments. The setup was thoroughly cleaned with soap and water between each session. The experimental setup was situated behind a light- and sound-proof curtain.

Animal were placed into a rear corner of the dark compartment and behavior was recorded for 5 min. Afterwards the animal was placed back into its home cage.

A trained observer blind to the experimental condition measured the time and entries into the light compartment by using customized freeware (EVENTLOG, Robert Henderson, 1986).

#### Startle response

Eight identical startle set-ups were used consisting of a non-restrictive Plexiglas cylinder (inner diameter 4 cm, length 8 cm) mounted onto a plastic platform. Every set-up was implemented in a sound attenuated chamber (SR-LAB, San Diego Instruments SDI, San Diego, CA, USA). The cylinder movement was detected by a piezoelectric element mounted under each platform. The voltage output was amplified and then digitized (sampling rate 1 kHz) by a computer interface (I/O-board provided by SDI). The peak voltage output within 50 ms after stimulus onset was taken as the startle amplitude. For quantification SR-LAB software was used. Before every run we calibrated response sensitivity to assure identical output levels of every chamber. Sounds were administered through a high-frequency speaker placed 20 cm above the cylinder. Control stimulus and three different startle stimuli were delivered: white noise bursts of 20 ms duration as control and 75, 90, 105, and 115 dB(A) intensity in a constant background noise of 50 dB(A) as startle stimuli.

Animals were placed gently into the cylinder. After an acclimation period of 5 min, 10 control trials and 20 startle stimuli of each intensity were presented in pseudorandom order. The interstimulus interval was 15 s on average (13–17 s, pseudorandomized). Plexiglas cylinders were cleaned thoroughly with soap water after each trial.

### Manganese-enhanced magnetic resonance imaging (MEMRI)

#### Scanning procedure

*In vivo* MEMRI was performed essentially as described before (Grünecker et al., 2010, 2012). Briefly, minimum 2 weeks after the last testing animals received intraperitoneal injections of 30 mg/kg MnCl_2_ (Sigma, Germany) every 24 h over the course of 8 consecutive days. This protocol (8 × 30/24 h) was found to balance systemic side effects and satisfy MEMRI contrast in an optimized manner (Grünecker et al., 2010). Further we could demonstrate that *in vivo* MEMRI is a valid tool to measure volume differences and has several advantages compared to *ex vivo* measurements, e.g., it avoids possible distortions during brain extraction steps and allows for normalization to the whole brain volume (Golub et al., 2011).

MEMRI experiments were performed on a 7T Avance Biospec 70/30 scanner (Bruker BioSpin, Ettlingen, Germany). Imaging took place 12–24 h after the last injection. Mice were anaesthetized with isoflurane (DeltaSelect, Germany) and fixed in a prone position on a saddle-shaped receive-only coil, where they were further kept under inhalation anesthesia with an isoflurane-oxygen mixture (1.5–1.7 vol% with an oxygen flow of 1.2–1.4 l/min). Head movements were prevented by fixing the head with a stereotactic devise and the frontal teeth with a surgical fiber. Body temperature was monitored with a rectal thermometer (Thermalert TH-5, Physitemp Instruments, USA) and kept between 34 and 36°C using a water-based heating pad. Pulse rate was continuously monitored by a plethysmographic pulse oxymeter (Nonin 8600V, Nonin Medical Inc., USA).

T_1_-weighted (T1w) brain images were acquired using a 3D gradient echo pulse sequence [TR = 50 ms, TE = 3.2 ms, matrix size = 128 × 106 × 106 zero filled to 128 × 128 × 128, field of view (FOV) = 16 × 16 × 18 mm^3^, number of averages = 10, resulting in a spatial resolution of 125 × 125 × 140.6 μm^3^ with a total measurement duration of 90 min]. Additionally, 3D T_2_-weighted (T2w) images were obtained using a RARE (rapid acquisition relaxation enhanced) pulse sequence (TR = 1000 ms, TE = 10 ms, matrix size = 128 × 112 × 112 zero filled to 128 × 128 × 128, FOV = 16 × 16 × 18 mm^3^, Number of averages = 2, Rare factor = 16, resulting in a resolution of 125 × 125 × 140.6 μm^3^, with a measuring time of around 30 min). Total measurement time was around 2 h. Animals were sacrificed after scanning.

#### MRI data post-processing

Images were reconstructed using Paravision software (Bruker BioSpin, Ettlingen, Germany) and transferred to standard ANALYZE format. Further post-processing was performed using SPM 8 (www.fil.ion.ucl.ac.uk/spm). All images were bias corrected to remove intensity gradients introduced by the geometry of the surface coil. A representative T2w image was selected that served as a first template where all T2w images were spatially normalized to. A group template was then produced based on an average of all normalized images. Bias corrected images of all individual animals were then normalized a second time to the group template. For brain extraction, normalization steps of T2w-images were carried out first due to their better contrast between parenchyma and other tissue types and no signal hyperintensity of large vessels compared to T1w-images. A binary mask defining the intracranial vault without large vessels (whole brain) was defined (MRIcro, www.sph.sc.edu/comd/rorden/mricro.html) on the T2w-group template, and transformed to native (co-registered) space of each individual animal (by inverted spatial normalization). Brain extracted images of the co-registered and bias corrected T1w-images were then used for the normalization steps of T1w-images.

#### Regions of interest (ROI) analysis of MEMRI contrast

ROIs were defined based on the anatomical atlas of the C57BL/6 mouse (Paxinos and Franklin, 2001). The binary whole brain mask from the brain-extraction step was back-transformed into native space of bias corrected raw T1w-images for each animal. Binary masks of the whole HPC as well as the ventral and the dorsal parts of each hemisphere separately were manually created on the bias corrected raw T1w-images due to the extreme morphological changes introduced by IA. The boundary between the dorsal and the ventral hippocampus was defined by anker points based on the T1w-images (50% of the dorso-ventral extension). Volume measurements of all ROIs were performed using an in-house written program in IDL (www.creaso.com). *Remaining volume* was defined as the ratio of the measured volume in IA mice and the mean volume of Veh animals in the same region. ROIs were specified by a task-trained scientist blind to the experimental condition. Reliability of manually defined ROIs was verified in a previous study by a high interrater correspondence of volume results for three task-trained raters (*r* > 0.95, *p* < 0.006). For 3D reconstruction of representative hippocampi we used an in-house written program in IDL.

### Statistical analysis

For analysis and presentation of all data we used GraphPad Prism 5.0 (GraphPad, San Diego, CA, USA), SPSS 16.0 (SPSS, Chicago, IL, USA), and MATLAB (MathWorks, Natick, MA, USA). Data are presented as mean ± SEM. A *p* ≤ 0.05 was accepted as statistically significant. The learning parameters accuracy, latency, wrong platform visits, and start bias were submitted to a One-Way ANOVA with repeated measurements to examine differences between the different learning protocols as well as improvement in learning over the course of days. The behavioral performance of week 1 and week 2 were separately analyzed. The number of non-accurate and accurate learners as well as the number of non-biased and biased starters on d5 or d5_r_ was contrasted between the different protocols by the χ^2^-test. Accuracy scores on d5 or d5_r_ were compared to a theoretical value of 100% by student's *t*-test. The distribution of the chosen arm in the probe trial of the 1st week was compared to the one of the 2nd week by a χ^2^-test. For open field, holeboard, and light-dark test, student's *t*-test for independent samples was applied on each parameter. Startle responses were compared with a Two-Way ANOVA [factor 1: intensities (INT), factor 2: treatment]. For the comparison of the travelled distance over time in the open field test, a Two-Way ANOVA for repeated measures was applied (within factor: time, between factor: treatment). Differences between IA and Veh group in HPC volume or accuracy were tested by student's *t*-test. For the comparison of the remaining HPC volume between learners (L) and non-learners (NL), IA animals were first assigned to one or the other group by the threshold of 83.3% accuracy on d5_r_ within free choice protocol. Student's *t*-test was then used to contrast the remaining HPC of both groups. Linear dependence of behavioral parameters and HPC volume was measured by Pearson product-moment correlation coefficient, and cases with missing data were excluded list wise.

## Results

### Place, but not response strategies enable for reversal learning

We first compared the efficiency of reversal learning protocols (Figure 1B). Three different learning modalities were tested independently in three groups of mice, i.e., place (P), response (R), and free choice (F) learning.

**Figure 2:**
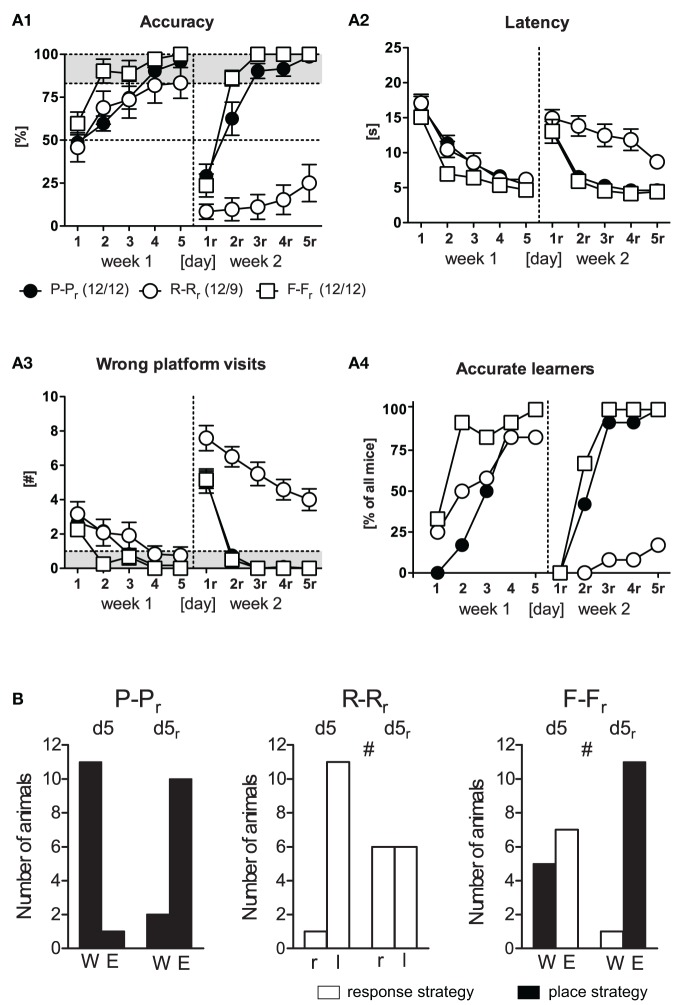
**Reversal learning.** Three groups (P-P_r_, R-R_r_, F-F_r_) underwent reversal learning with a probe trial in the end of each week. **(A)** In the 1st week, spatial learning occurred in all groups equally represented in all four learning parameter. In the 2nd week, only the accurate learners of week 1 of the R-R_r_ group, but not of the P-P_r_ and the F-F_r_ groups, were unable to acquire the new platform position, as evident from decreased accuracy as well as increased latency and wrong platform visits [*Protocol: F*_(2, 30)_ ≥ 22.09, *p* < 0.001, *Protocol × Day: F*_(8, 120)_ ≥ 2.86, *p* ≤ 0.006]. Merely 2/9 animals of the R-R_r_ group reached the accurate learner criterion on d5_r_. **Performance parameters: (A1)** Accuracy, expressed as the percentage of accurate trials per day. An accurate trial was defined as direct swimming to the platform without entering another arm before **(A2)**. Latency until mice climbed onto the hidden platform, averaged over the 6 trials per day **(A3)**. Number of wrong platform visits, defined as the visits to the outer third of the wrong arm averaged per day **(A4)**. Percent of accurate learners, that is accomplishing ≥83% of accurate trials per day (≥5 out of 6 trials). **(B)** Number of animals assigned to either allocentric or egocentric searching strategy according to the probe trial at the end of each week. Deviant distributions were observed in the R-R_r_ group representing performance by chance in the 2nd week and in the F-F_r_ group representing a switch to allocentric strategy in week 2 (χ^2^ ≥ 5.04, *p* ≤ 0.024, ^#^*p* ≤ 0.05).

In the 1st week of training, mice readily acquired the WCM task irrespective of the training protocol (Figures 2A1–A4, d1–d5). This became evident by an increase in accuracy and a decrease in latency and wrong platform visits over *Days* [*F*_(4, 132)_ ≥ 25.54, *p* < 0.001] with no main effects of the *Protocol* [*F*_(2, 33)_ ≤ 2.83, *p* ≥ 0.073] and no *Day × Protocol* interaction [*F*_(8, 132)_ ≤ 1.39, *p* ≥ 0.204]. If the levels of accuracy shown at d5 were considered, most of the mice had successfully reached the accuracy criterion of ≥5 accurate out of 6 trials (place training: 12/12, response training: 9/12, free training: 12/12; Figure 2A4). Noteworthy, the three mice of the response training group that failed to reach the accuracy criterion did not perform at random, but adopted a different response to find the platform (turn right into the wrong arm, turn around and swim straight ahead to the platform at the end of the opposite arm, instead of turning left, as evident from accuracy scores ≤ 17%). The parallel increase in accuracy and the number of accurate learners suggests rather an increase in the number of accurate learners (indicating a light bulb effect) then a stable rise in accuracy in each mouse on each day.

During the 2nd week, the accurate learners of week 1 underwent reversal learning by relocation of the platform to the opposite arm. At the first day of relearning, all mice showed memory perseverance, as reflected by the high number of visits to the original platform position (i.e., wrong platform visits) and the resulting increase in escape latencies and the low levels of accuracy. Ongoing place and free choice training led to rapid relearning of the new platform position. In contrast, there was virtually no relearning in the response training group (Figure 2A, d1_r_–d5_r_). This was reflected by significant main effects of *Protocol* [*F*_(2, 30)_ ≥ 22.09, *p* < 0.001] and *Protocol × Day* interactions [*F*_(8, 120)_ = 2.86, *p* ≤ 0.006] in accuracy, latency, and wrong platform visits. The 3–7 wrong platform visits on d5_r_ were indicative of a remarkably perseverance of the originally learned platform position in the response learning group. Only 2/9 animals successfully acquired the new platform position at d5_r_ (compared to 12/12 mice undergoing place or free choice learning; Figure 2A4). Taken together these data demonstrate that C57BL6/N mice learned equally well to locate the platform position during the 1st week of training irrespective of the training protocol. During the 2nd week, however, the mice failed to accurately learn the new platform position upon response training.

The probe trials performed at the end of training on d5 and d5_r_ were analyzed in terms of the number of animals that had turned into one or the other arm (Figure 2B). Upon place learning, almost all animals entered the arm that contained the platform during training. Deviant distributions were observed in the response learning group on d5_r_ resembling performance by chance in the 2nd week (χ^2^ = 5.04, *p* = 0.024). On d5, free choice learners started for the first time from a new position (North arm). Nearly equal numbers adopted an place (5/12) vs. response (7/12) strategy. After the 2nd week of training, however, 11/12 animals adopted a place strategy (χ^2^ = 6.75, *p* = 0.009). These data indicate that C57BL6/N mice can successfully acquire the platform position upon free choice training by acquiring either a place or a response. Accurate relearning of the platform position, in contrast, favors place learning strategies.

### Relearning capabilities are independent of the original learning strategy

**Figure 3:**
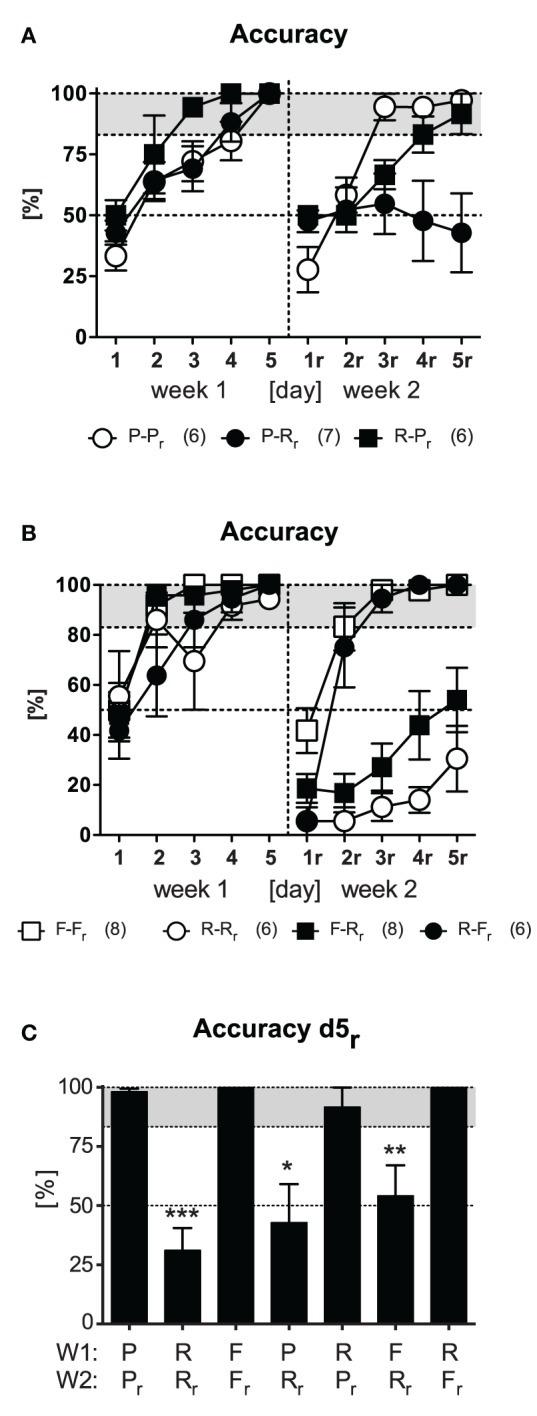
**Strategy switching.** Four strategy switching protocols (P-R_r_, R-P_r_, F-R_r_, R-F_r_) were used (cf. Figure 1B). Reversal protocols (F-F_r_, R-R_r_) were carried as control and replication. **(A)** After accurate learning in week 1, only animals that underwent the P-R_r_ protocol in the 2nd week failed to learn the task [exemplarily shown for accuracy; *Protocol: F*_(2, 16)_ = 3.24, *p* = 0.066, *Protocol × Day: F*_(8, 64)_ = 8.14, *p* < 0.001]. 2/7 animals performed accurate on d5_r_. **(B)** After accurate learning in the 1st week, only those animals which underwent F-R_r_ training were unable to acquire the task in the 2nd week [exemplarily shown for accuracy; *Protocol: F*_(3, 24)_ = 83.35, *p* < 0.001, *Protocol × Day: F*_(12, 96)_ = 5.28, *p* < 0.001]. 3/8 animals reached the accurate learner criterion. **(C)** Accuracy on d5_r_ is shown for all protocols [1st week (W1), 2nd week (W2)]. Animals failed to acquire the task upon response relearning in the 2nd week [*t* ≥ 3.52, *p* ≤ 0.012 (one sample *t*-test); *p* ≤ 0.05^*^, *p* ≤ 0.01^**^, *p* ≤ 0.001^***^].

We next investigated whether the deficits in reversal learning upon response training reflect the general inability of switching from an established response pattern to any other platform position. To this end we trained new groups of animals as described before (Figure 1B) but switched between response and place learning protocols (strategy switching; P-R_r_, R-P_r_). As a control and replication, a reversal place learning group was included (P-P_r_). After animals easily learned the tasks in the 1st week [*Day: F*_(4, 64)_ ≥ 36.78, *p* < 0.001 in accuracy, latency, and wrong platform visits; for the sake of clarity and brevity here and hereafter only accuracy is shown in Figures 3A,B], only the group of animals that switched to the response learning protocol failed to successfully acquire the new platform position in the 2nd week as reflected by a trend for *Protocol* [*F*_(2, 16)_ = 3.24, *p* = 0.066] and an significant *Protocol × Day* interaction [*F*_(8, 64)_ = 8.14, *p* < 0.001]. These animals again showed remarkably perseverance of the old platform position, as indicated by an increased latency and high numbers of wrong platform visits [*Protocol: F*_(2, 16)_ ≥ 4.21, *p* ≤ 0.034; *Protocol × Day: F*_(8, 64)_ = 3.47, *p* < 0.002]. Merely 2/7 animals reached the accurate learner criterion on d5_r_ after being switched from place to response training as opposed to 5/6 mice switching from response to place learning (χ^2^ = 3.89, *p* = 0.048).

In a second experiment with new groups of animals, we contrasted response and free choice learning protocols by strategy switching (F-R_r_, R-F_r_). This time, a reversal response (R-R_r_) and free choice learning group (F-F_r_) were used as controls. After successful acquisition in the 1st week of training, only animals that underwent response training failed to be accurate in the 2nd week and kept swimming to the old platform position [i.e., R-R_r_, F-R_r_; *Protocol: F*_(3, 24)_ ≥ 24.63, *p* < 0.001 in accuracy, latency, and wrong platform visits; Figure 3B]. Only 3/8 animals that switched from free choice to response protocol reached the accurate learner criterion as opposed to 6/6 mice which switched from response to free choice learning (χ^2^ = 5.83, *p* = 0.015).

We next questioned whether extensive response reversal training or intermittent free choice training prior to response reversal training would enable reversal of the initial response. To this end, we continued the training with two groups of the 2nd experiment (R-R_r_, R-F_r_) for another week, whereby both groups underwent response protocol based on right turns (same protocol as for R_r_, i.e., R-R_r_-R_r_, R-F_r_-R_r_). Despite successful relearning under free choice protocol in the 2nd week, none of the two conditions enabled for response relearning in the 3rd week (data not shown). Remarkable was that only one animal of each group exceeded the accurate learner criterion in the last week. This experiment demonstrates that mice were unable to relearn a response even upon extensive training (R-R_r_-R_r_) or after they might have acquired a new response upon successful free training (R-F_r_-R_r_).

Summarizing all results obtained so far, animals failed to acquire the task upon response relearning as indicated by significant accuracy reduction on d5_r_ (*t* ≥ 3.52, *p* ≤ 0.012; Figure 3C) irrespective of the learning protocol employed in the 1st week. There was no compensatory effect of extensive training. In contrast mice readily acquired the new platform position upon place training. The same was the case for the free choice protocol. The fact that most of these animals used a place strategy during the probe trial in the end of the 2nd week (cf. Figure 2B, F-F_r_) supported the notion of the superiority of place learning vs. response learning during relearning of the platform position.

### The HPC enables for place learning and relearning

**Figure 4:**
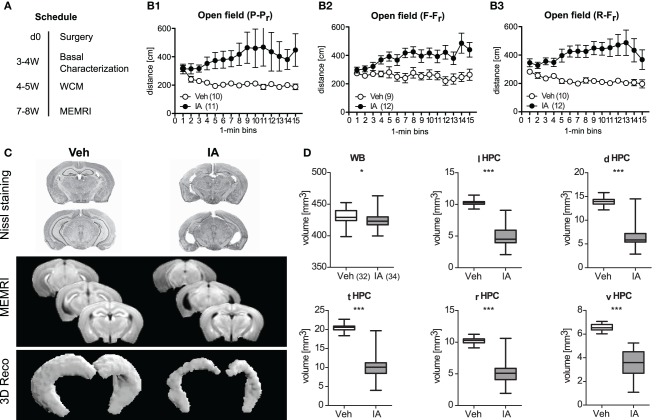
**Consequences of ibotenic acid lesions. (A)** Schedule after surgery [d (day), week (W); for a summary of the results of the basal characterization see Table 1]. Panel **(B1–B3)** There were three independent batches of sham-operated (Veh) or ibotenic acid-lesioned (IA) mice. Veh controls habituated to the open field or at least stayed at a constant exploration level, whereas IA mice even showed a sensitized response in a highly replicable manner among three cohorts of mice (*Treatment: F* ≥ 5.48, *p* ≤ 0.029; *Treatment × Time: F* ≥ 2.09, *p* ≤ 0.012). **(C)** *In vivo* manganese-enhanced magnetic resonance imaging (MEMRI) was used for volumetry of the hippocampus (HPC) subregions. Representive Nissl stained slices, mean T1-weighted images and representive 3D reconstructions of the HPC for Veh and IA animals are depicted. **(D)** Injection of IA into the HPC was sufficient to reduce its volume in total and in all subregions [total hippocampus (_t_HPC), left hippocampus (_l_HPC), right hippocampus (_r_HPC), dorsal hippocampus (_d_HPC), ventral hippocampus (_v_HPC), *t*_64_ ≥ 16.55, *p* < 0.001]. A less pronounced reduction was also observed for the whole brain (WB) volume (*t*_64_ = 13.60, *p* = 0.017, ^*^p ≤ 0.05, ^***^p ≤ 0.001).

Given the well-known role of the HPC in place learning, we investigated the importance of the HPC for relearning capabilities. In three independent cohorts of animals, bilateral lesions of the HPC were precipitated by IA and opposed to consequences in sham-treated controls. The mice were tested for consequences of the lesions on exploration, locomotion, anxiety-like behavior, and acoustic startle responses 3–4 weeks after surgery (Figure 4A). IA mice showed less immobility time, longer travel distances, and less rearing duration consistent, for the open field and holeboard test (*t* ≥ 4.31, *p* < 0.001; Table 1). The number of rearings was not affected. This hyperactivity failed to affect anxiety-related behavior in the light-dark test and acoustic startle responses (Table 1). A more closer look at the temporal development of locomotor activity in the open field revealed a common picture among the three batches of mice: horizontal locomotion started at the same level in IA animals as Veh, but increased with time (sensitization) in IA mice, whereas Veh controls stayed at the same level or even decreased (*Treatment: F* ≥ 5.48, *p* ≤ 0.029; *Treatment × Time: F* ≥ 2.09, *p* ≤ 0.012; Figures 4B1–B3).

**Table 1:** **Consequences of HPC lesions on basic exploration and anxiety-related behavior**.

**Test**	**Parameter**	**Sham**	**HPC lesion**	**Statistics**
Open field	**Immobility time**	383 ± 10 s	287 ± 11 s	*t*_(69)_ = 6.333, ***p* < 0.001**
	**Distance**	3463 ± 133 cm	5887 ± 430 cm	*t*_(69)_ =5.068, ***p* < 0.001**
	Rearing (number)	252 ± 9	258 ± 10	*t*_(69)_ = 0.488, *p* = 0.627
	**Rearing (duration)**	327 ± 13 s	250 ± 12 s	*t*_(69)_ = 4.306, ***p* < 0.001**
	**Center time**	36 ± 2%	27 ± 2%	*t*_(69)_ = 3.180, ***p* = 0.002**
	**Center distance**	36 ± 2%	25 ± 2%	*t*_(69)_ = 4.433, ***p* < 0.001**
Holeboard	**Immobility time**	533 ± 12 s	354 ± 18 s	*t*_(64)_ = 7.836, ***p* < 0.001**
	**Distance**	3586 ± 93 cm	7492 ± 649 cm	*t*_(64)_ = 5.446, ***p* < 0.001**
	Rearing (number)	246 ± 10	246 ± 13	*t*_(64)_ = 0.011, *p* = 0.992
	**Rearing (duration)**	362 ± 20 s	239 ± 16 s	*t*_(64)_ = 4.876, ***p* < 0.001**
	Nose pokes	33 ± 3	32 ± 2	*t*_(64)_ = 0.374, *p* = 0.709
	Nose pokes (duration)	21 ± 2 s	17 ± 1 s	*t*_(64)_ = 1.715, *p* = 0.091
Light-dark	Time in light	31 ± 2%	35 ± 2%	*t*_(69)_ = 1.180, *p* = 0.242
	Light entries	42 ± 1%	42 ± 1%	*t*_(69)_ = 0.149, *p* = 0.882
Startle responses	Background	17 ± 1mV	17 ± 1mV	*Two-Way ANOVA:*
	75dB	34 ± 4mV	33 ± 2mV	– intensity × treatment
	90dB	58 ± 11mV	57 ± 8mV	*F*_(4, 280)_ = 0.733,
	105dB	264 ± 29mV	277 ± 27mV	*p* = 0.990 – Treatment
	115dB	393 ± 28mV	392 ± 34mV	*F*_(1, 70)_ = 0.012, *p* = 0.915

Data are shown as mean ± SEM of n = 33 Veh and n = 38 IA mice deriving from all three batches (P-P_r_, F-F_r_, R-F_r_).

p values ≤ 0.05 are accepted as significant and written in bold.

The success of IA lesions was confirmed by means of *in vivo* MEMRI performed in the end of the experiment (i.e., 7–8 weeks after surgery; Figure 4A). We used this *in vivo* approach since *ex vivo* techniques are often accompanied by volume shrinkage (Golub et al., 2011). IA led to a slight reduction in whole brain volume [by 2% on average; *t*_(64)_ = 13.60, *p* = 0.017] and a large reduction in total HPC volume (by ~50%), which was comparable for the left/right and dorsal/ventral HPC [*t*_(64)_ ≥ 16.55, *p* < 0.001; Figures 4C,D].

**Figure 5:**
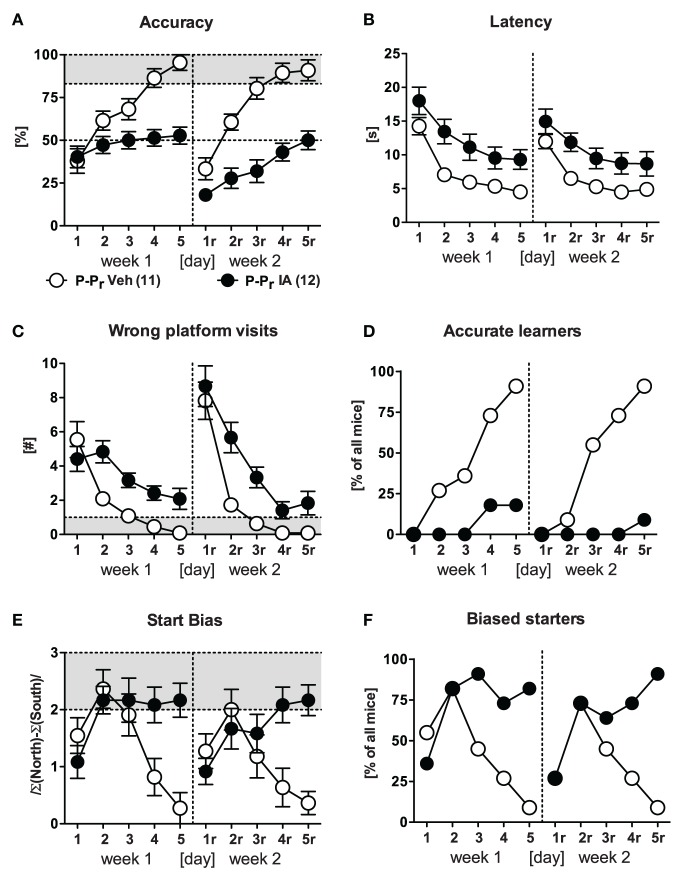
**Place learning in HPC-lesioned animals.** Veh and IA mice underwent the P-P_r_ protocol. **(A,D)** IA mice could not perform accurately during learning and relearning phase [*Treatment: F*_(1, 21)_ ≥ 14.32, *p* ≤ 0.001, *Treatment × Day: F*_(4, 84)_ ≥ 5.17, *p* ≤ 0.001]. Only 2/12 in the 1st and 1/12 in the 2nd week reached the accurate learner criterion on d5_r_. **(B,C)** IA mice showed longer latencies and more wrong platform visits in both weeks [*Treatment: F*_(1, 21)_ ≥ 6.61, *p* ≤ 0.008]. Noteworthy IA group learned to reduce wrong platform visits over time which was indicated by the parallel regress in both groups. **(E,F)** The high start bias and the high number of bias starters at the end of each week indicated that the IA animals developed an alternative response to solve the task (for details see the text).

The first batch of IA and Veh animals underwent place training for learning and relearning after completion of the basal characterization (i.e., 4–5 weeks after surgery; Figures 5A–F). IA animals failed to acquire the task in both weeks [*Treatment: F*_(1, 21)_ ≥ 14.32, *p* ≤ 0.001; *Treatment × Day: F*_(4, 84)_ ≥ 5.17, *p* ≤ 0.001; Figure 5A]. Merely 2/12 IA mice in the 1st and 1/12 in the 2nd week achieved the accurate learner criterion (Figure 5D) as compared to 10/11 and 11/11 Veh controls (χ^2^ ≥ 12.68, *p* < 0.001). It is noteworthy that both groups of mice showed a comparable decrease in escape latencies over the course of training during learning and relearning [*Day: F*_(4, 84)_ ≥ 38.57, *p* < 0.001; *Treatment × Day: F*_(4, 84)_ ≤ 0.87, *p* ≥ 0.480], but starting from a different level [*Treatment: F*_(1, 21)_ ≥ 6.61, *p* ≤ 0.018; Figure 5B]. In line with the decreased latencies was the regress in wrong platform visits during learning and, even more pronounced, during relearning [*Day: F*_(4, 84)_ ≥ 17.94, *p* < 0.001; *Day × Treatment: F*_(4, 84)_ ≥ 4.03, *p* = 0.005 for the 1st week]. Again the treatment effect was still present [*Treatment: F*_(1, 21)_ ≥ 13.37, *p* = 0.001; Figure 5C]. At the same time, accuracy levels of IA mice had barely surpassed the chance level of 50%, suggesting random performance of these animals. However, on closer inspection of the data it became evident that the majority of the mice developed a clear turn-bias: Animals performed the same turn, left or right, irrespective of the starting position. Still, mice could discriminate between the arms, as they swam straight ahead in the correct arm or performed an U-turn before the end of the wrong arm. This led to a bimodal distribution of accuracy depending on the starting position (100 vs. 0% accuracy). Quantification of this start bias confirmed our observations (Figures 5E,F). In the beginning of training, both IA and Veh animals seemed to acquire a response strategy, which was characterized by an increased start bias. Upon ongoing training, Veh controls switched to place learning, as indicated by a decrease in start bias, whereas IA mice consistently remained on high levels [*Day: F*_(4, 84)_ = 5.185, *p* = 0.001; *Treatment: F*_(1, 21)_ = 4.547, *p* = 0.045; *Treatment × Day: F*_(4, 84)_ = 5.966, *p* < 0.001]. This was also reflected by the percentage of biased starters of the IA group (82% on d5 and 91% on d5_r_ as opposed to 9% on d5 and 9% on d5_r_ of Veh; χ^2^ ≥ 12.68, *p* < 0.001). The development of a response-based strategy explains the decreases in wrong platform visits and escape latencies over the course of training.

**Figure 6:**
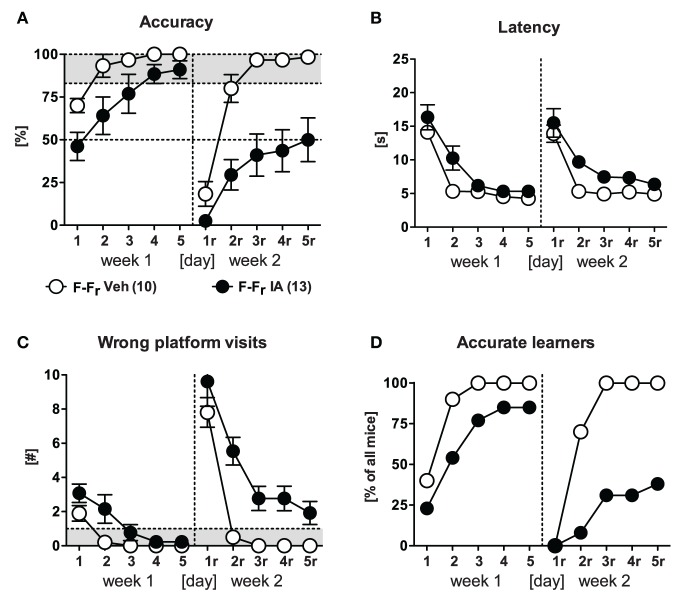
**Free choice learning and relearning in HPC-lesioned animals.** IA mice and Veh controls underwent free choice learning in the 1st week and free choice learning in the 2nd week (F-F_r_). **(A,D)** IA mice could not acquire the free choice task in the 2nd week as shown by low accuracy values [*Treatment: F*_(1, 21)_ = 20.02, *p* < 0.001] and low numbers of accurate learners (5/13). **(B,C)** In the 2nd week IA mice displayed longer latencies and more wrong platform visits [*Treatment: F*_(1, 21)_ ≥ 7.88, *p* ≤ 0.011]. Again there was a parallel decrease observed in both parameters indicating the acquisition of an alternative response.

The second batch of IA and Veh animals underwent free training during both learning and relearning of the platform position (Figures 6A–D). IA animals were mildly impaired in learning during the 1st week. This became evident by decreased accuracy and increased wrong platform visits [*Treatment: F*_(1, 21)_ ≥ 5.24, *p* ≤ 0.032] but no differences in escape latency. Still both groups could readily acquire the task until d5 which was resembled by 11/13 accurate learners in the IA group compared to all (10/10) in the Veh controls. During the 2nd week, however, IA mice failed to learn the new platform position. This became apparent by a significant *Treatment* effect in accuracy, latency, and wrong platform visits [*F*_(1, 21)_ ≥ 7.88, *p* ≤ 0.011]. Only 5/13 animals reached the accurate learner criterion opposed to 10/10 Veh controls (χ^2^ = 9.43, *p* = 0.002). Noteworthy that IA mice were able to reduce escape latencies and the wrong platform visits parallel to Veh animals in the 2nd week [*Day: F*_(4, 84)_ ≥ 33.36, *p* < 0.001; *Day × Treatment: F*_(4, 84)_ ≤ 3.27, *p* ≥ 0.122], thus confirming the results obtained in IA mice which underwent place learning (Figures 5B,C).

**Figure 7:**
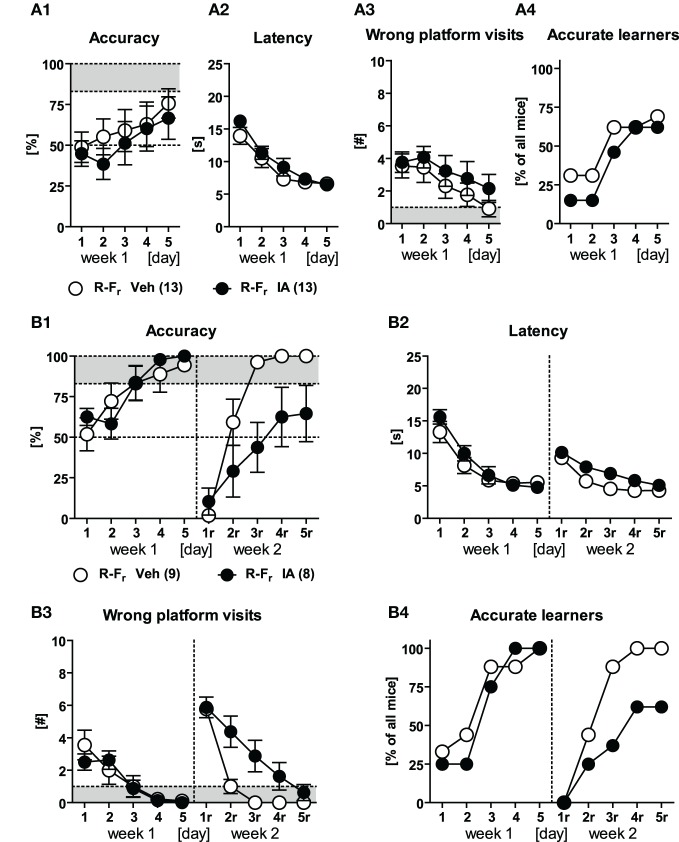
**Response learning and free choice relearning in HPC-lesioned animals.** Veh and IA animals underwent response learning in the 1st week and free choice learning in the 2nd week (R-F_r_). **(A1–A4)** Mice from both treatment groups performed equally well upon response training. However both groups failed to reach the accuracy criterion because 4/13 Veh and 5/13 IA animals had adopted a “wrong” response indicated by accuracy levels below 17%. Therefore, we additionally cleared the data from these animals and re-examined training performance for both weeks. **(B1–B3)** IA mice had no impairments in response learning in the 1st week but showed a reduced accuracy and a higher latency as well as more wrong platform visits in the 2nd week [*Treatment: F*_(1, 15)_ ≥ 5.25, *p* ≤ 0.036]. However animals of both groups reduced the latency and the wrong platform visits over time in the 2nd week [*Day: F*_(4, 60)_ ≥ 47.65, *p* < 0.001]. **(B4)** Until d5_r_, 5/9 IA mice still reached the accurate learner criterion in the 2nd week as opposed to 9/9 Veh controls (cf. Figure 8D).

The third batch of IA and Veh animals passed through the response protocol in the 1st week (to assess the effects of HPC lesions on this learning strategy) and the free choice protocol in the 2nd week (to confirm the findings of the 2nd batch). During training in the 1st week, there were no group differences in accuracy, latency, number of wrong platform visits, and the number of accurate learners [Figure 7A; *Treatment: F*_(1, 24)_ ≤ 0.92, *p* ≥ 0.346; *Day × Treatment: F*_(4, 96)_ ≤ 0.87, *p* ≥ 0.485]. However, a considerably high number of animals did not exceed the accurate learner criterion independently of the treatment (Figure 7A4). These animals adopted a “wrong” response by turning right followed by a U-turn and swimming ahead instead of just turning left (indicated by accuracy scores below 17%). For that reason five animals from the IA group and four from the Veh group were excluded from data re-analysis (Figure 7B). The remaining IA animals were able to learn the response similarly well as the remaining Veh controls [*Treatment: F*_(1, 15)_ ≤ 0.63, *p* ≥ 0.441; *Day × Treatment: F*_(4, 60)_ ≤ 1.25, *p* ≥ 0.299]. However, they showed reduced accuracy levels in the 2nd week as well as higher escape latencies and more wrong platform visits [*Treatment: F*_(1, 15)_ ≥ 5.25, *p* ≤ 0.036; Figures 7B1–B3 d1_r_–d5_r_]. Nevertheless both groups learned to reduce the latency and the wrong platform visits over time while relearning [*Day: F*_(4, 60)_ ≥ 47.65, *p* < 0.001]. 9/9 Veh animals but still 5/8 IA mice reached the accurate learner criterion in the 2nd week. This forced us to work on the relationship between the dimension of the HPC lesion and the ability of the mice to relearn the platform position upon free choice learning (see next section).

We conclude from the experiments that IA animals failed to accurately locate the platform upon place training, but were still able to reduce their latency and wrong platform visits over the course of training without having an intact HPC. This could be explained by an alternative response learning described with the start bias. IA animals that underwent free choice learning and had the choice between place and response strategy readily learned the platform position in the 1st week, because they could employ HPC-independent response learning. The animals' performance dropped down during relearning in the 2nd week because of their failure to adopt HPC-dependent place learning.

### Residual volume of the left ventral HPC reflects relearning differences

**Figure 8:**
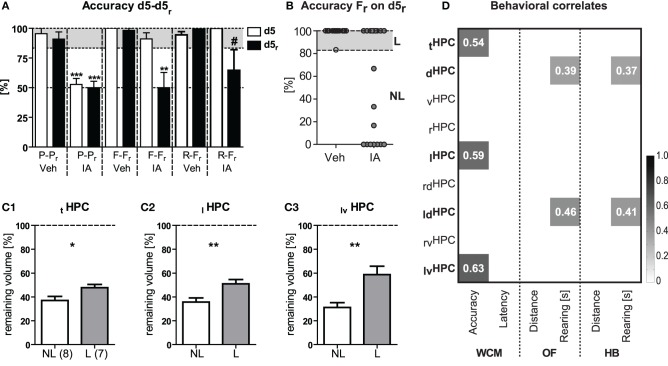
**Relearning performance and HPC shrinkage.** HPC volume reduction following ibotenic acid treatment was related to distinct behavioral phenotypes. **(A)** Accuracy at d5 and d5_r_ is shown for all three batches of animals (cf. Figures 5–7). IA animals failed to learn the platform position under P-P_r_ protocol [*t*_(11)_ ≥ 9.21, *p* < 0.001]. For F-F_r_ protocol, IA mice accurately learned the task in the 1st but failed in the 2nd week [*t*_(12)_ = 3.91, *p* = 0.002]. IA mice that underwent R-F_r_ tended to be unable to acquire the new platform position in the 2nd week [*t*_(7)_ = 2.03, *p* = 0.081]. The latter could be related to less severe lesions. **(B)** IA mice from batches 2 to 3 undergoing F_r_ relearning were assigned to learners (L) and non-learners (NL) according to their accuracy scores on d5_r_. **(C1–C3)** Subsequent re-analysis of HPC volume changes in NL and L revealed highest differences in the remaining volume for the ventral portion of the left HPC [*t*_(13)_ = 3.52, *p* = 0.003] followed by the left HPC in total [*t*_(13)_ = 3.02, *p* = 0.009]. This had still an effect on total HPC volume [*t*_(13)_ = 2.39, *p* = 0.032]. **(D)** HPC volume of different portions in the HPC was correlated with the learning performance of those IA animals. Analysis confirmed a significant relationship between the averaged accuracy of week 2 (i.e., F_r_ training) and the left ventral portion of the HPC, the total left HPC as well as total HPC [correlation (Pearson's r) depicted in grey value and explicitly advertised in each quadrant; *p* ≤ 0.05]. No similar links were seen for the averaged escape latencies. This indicates a crucial role particular of the left ventral HPC for relearning of the platform position and suggests accuracy rather than escape latency as a valid measure of HPC-dependent learning. Correlation of open field (OF) as well as holeboard (HB) behavior and HPC volume point to an important contribution of the dorsal and in particular the left dorsal HPC to rearing duration (under implication of all IA and Veh mice). ^*^p ≤ 0.05, ^**^p ≤ 0.01, ^***^p ≤ 0.01, ^#^*p* ≤ 0.1.

The consequences of HPC lesions on accuracy at d5 and d5_r_ were summarized in Figure 8A. IA mice from the 2nd and 3rd batch showed a remarkable variance in accuracy upon free choice re-training on d5_r_. This became evident by a clear bimodality in the performance of the animals (Figure 8B). In order to identify potential relationships between the remaining volume of the HPC and relearning capabilities, we assigned free choice retrained IA mice to learners and non-learners on the basis of their accuracy on d5_r_ (Figure 8B). Compared to the learners, non-learners showed the most pronounced volume reduction in the ventral portion of the left HPC [*t*_(13)_ = 3.52, *p* = 0.003; Figure 8C3] followed by the left HPC in total [*t*_(13)_ = 3.02, *p* = 0.009; Figure 8C2]. This had still an effect on the total HPC volume [*t*_(13)_ = 2.39, *p* = 0.032; Figure 8C1]. No similar differences could be observed for the left dorsal [*t*_(13)_ = 1.92, *p* = 0.078] or the right HPC [right HPC: *t*_(13)_ = 1.13, *p* = 0.279; right ventral HPC: *t*_(13)_ = 0.42, *p* = 0.683; right dorsal HPC: *t*_(13)_ = 1.46, *p* = 0.1691]. Correlation analyses between the remaining volume of the different parts of the HPC and the mean accuracy and the mean latency of the 2nd week of IA mice confirmed a significant relationship between accuracy and the ventral part of the left HPC, the left HPC and the HPC in total (*p* ≤ 0.05; left ventral HPC: *r* = 0.63; left HPC: *r* = 0.59; total HPC: *r* = 0.54; Figure 8D). Remarkably, we failed to observe correlations between HPC volume and escape latencies. In contrast to relearning capabilities, correlation of the open field, or holeboard behavior and the HPC volume revealed significant correlations of the dorsal and, in particular, the left dorsal HPC (dorsal HPC: *r* ≥ 0.37; left dorsal HPC: *r* ≥ 0.41; *p* ≤ 0.05; Figure 8D) with total rearing time in IA mice.

In summary, highest functional contribution to relearning performance can be ascribed to the ventral part of the left HPC. Moreover, it is indicated that the dorsal HPC and, in particular, the left dorsal part contributes to basic exploratory behavior.

## Discussion

C57BL6/N mice that underwent reversal learning of place and free choice protocols could readily learn the new platform position. In contrast, mice trained with the response protocol were severely impaired in reversal learning. Even after extensive retraining for 2 weeks, these animals still performed around chance level and adhered to the original response-based strategy. Yet, they could easily overcome the initial response-based behavior and reach high levels of accuracy if relearning employed a place strategy, such as in the place and free choice protocol. In general, the mice were unable to successfully relearn the platform position, irrespective of the initial learning strategy, if relearning was based on a response. Together, these data implied that relearning and therefore spatial flexibility was successful only if the mice could use a place strategy to acquire the new platform position. By means of IA lesions we could demonstrate that such place relearning critically depends on an intact HPC.

### Advantages of water-cross maze testing

We could show that the WCM is a learning task for mice, which bears the possibility to clearly dissect response from place learning (Essman and Jarvik, 1961) and to differentially describe the learning success via distinct variables. The simplicity of the WCM leads to short trial durations and therefore reduces the stress load. This is reflected by the balanced use of place vs. response strategies in the probe trial after 1 week of free choice training (Packard and Wingard, 2004). A high stress load would be expected to favor response on the expenses of place learning (Schwabe et al., 2010). Most cross-maze tasks (e.g., T-maze) used food rewards to motivate the animals. This, however, requires food restriction, which represents a considerable stressor to the animals (Cabib et al., 2000). Another major advantage of water as a motive force is the more accurate and robust performance of the animals compared to food rewards (Ormerod and Beninger, 2002). All together, the WCM combines the benefits of its dry counterparts, which is the selective reinforcement of strategies and a direct measurement of the accuracy, with those of the Morris Water Maze, namely the water-based motivation and the absence of olfactory guidance by intramaze cues. Other than the retrospective and complex analysis of learning strategies in the Morris Water Maze task (cf. Wolfer and Lipp, 2000; Garthe et al., 2009), the WCM task allows for the simple assessment of the learning strategy online from the first day of training on by means of the standard parameters accuracy and start bias scores. In contrast, the main and often unique readout for the Morris Water Maze task is the escape latency (or escape distance). These measures, however, are affected by various non-cognitive factors such as motor impairments or motivation.

### Spatial flexibility requires place learning strategies

Most of the mice performed similarly well during acquisition of a place and a response. Nevertheless, there were a few non-learners under the response protocol, matching observations that place learning is easier than response learning for rats in the presence of prominent extramaze cues (Tolman et al., 1946). However, most of our mice showing inaccurate performance in the response protocol did not perform at random. Instead they had adopted an alternative response (taking a wrong turn first and then turn around to swim straight ahead to the platform in the opposing arm). Moreover, the side bias under place learning showed a strong increase between day 1 and day 2 before dropping down. This speaks for an acquisition of a response strategy before the place strategy comes into play (Figures 5E,F). Similar has been observed in the Morris Water Maze, when mice or rats had to locate a platform via environmental cues: before they acquired a place strategy, they stick to response-based navigation (Wolfer and Lipp, 2000; Harvey et al., 2008; Garthe et al., 2009). The most efficient learning occurred in mice that could employ both strategies and did not have to adjust to different starting positions, namely the free choice groups.

In contrast to memory acquisition during learning, there were clear differences upon reversal learning of the platform position. Animals trained with the response protocol performed poorly, while mice trained with the place or free choice protocol successfully learned the new platform position. Even ongoing relearning with the response protocol for one additional week did not improve the performance of the mice. This represents a largely unrecognized phenomenon, which, to the best of our knowledge, has not been explicitly demonstrated for mice. Ragozzino et al. (1999) found opposing results, as their intact control rats were able to reverse a response, yet, required considerably more trials than for a place reversal. Oliveira et al. (1997) demonstrate results similar to our findings as their control rats are severely delayed at response reversal and hippocampal lesioned rats exhibit constant impaired through the entire reversal phase. McDonald et al. (2001) report that control rats are incapable to succeed at reversal training in the same learning context—even after the double number of trials as for acquisition. Remarkably, the poorly performing mice of our study showed no signs of confusion, but instead a persistence of the original response-based strategy. This was reflected by the constantly high levels of perseverance errors (i.e., the number of wrong platform visits during relearning). Instead of performing at chance level of 50% accuracy, animals scored well below chance with an accuracy of only 20%. The most parsimonious explanation is that animals simply extended their original response-based strategy (swim left) to a slightly more complex one during relearning (swim left, turn around, swim ahead) due to constant reinforcement. We cannot rule out that relearning would be possible if the platform is inaccessible upon wrong choices (i.e., without reinforcement). In the present more naturalistic situation, where both options are given at any time, mice were not able to suppress their originally learned response. This indicates a higher rigidity compared to the other protocols and the one described in the rat literature.

We next investigated the ability of the mice to switch to another strategy in the 2nd week. It became evident that the animals could only successfully relearn the platform position if they were retrained with the place or free choice protocol. This was achieved independently of the original training protocol employed in the 1st week. Again, these findings seem to oppose those reported for rats passing through a strategy switch in a dry maze (Ragozzino et al., 1999). Rats were able to relearn under response protocol, yet it took them almost twice as much trials compared to rats undergoing place training in the 2nd week. Once again this point to a lower flexibility of C57BL6/N mice subjected to a response protocol.

Given the fact that free choice training allows for place and response learning at the same time, we hypothesized that relearning of the platform position was only possible if the animals would rely on place learning. This hypothesis was supported by the results of the probe trials performed after free choice learning in the 1st week and reversal learning in the 2nd week. During the probe trials, these animals started from the North arm for the first time. Through this, the choice to enter the East arm (left turn) or West arm (right turn) was indicative of the strategy the mice had chosen to locate the platform in the end of training. After the 1st week of training, approximately equal numbers of animals had acquired a place vs. a response strategy. This speaks in favor of comparable complexity of the strategies as well as a low stress level (Packard and Wingard, 2004; Elliott and Packard, 2008; Schwabe et al., 2010). Studies in rats report similar distributions after parallel acquisition of cued and spatial learning in the Morris water maze (McDonald and White, 1994; Sutherland et al., 1997). In contrast to the learning phase in the WCM, almost all mice located the platform with the help of a place strategy after the relearning phase. This finding strongly supported a crucial role of place strategies for spatial flexibility, but did not prove that they were essential. This was assessed with the help of HPC lesions described within the next section.

Comparing our data with the rat literature, mice and rats seem to acquire the two strategies similarly well and both species precede place learning with response learning during the course of place training. Contrary to the rat literature, our mice could not overcome an initially learned response even after extensive training in the WCM. This indicates that C57BL6/N mice adhere rigidly to the originally learned response whereas rats seem to overcome such response a lot easier and behave therefore more flexible. Yet, future research is needed to evaluate the possible role of stress as a driving force behind the inflexibility in mice [for rats see Engelmann et al. (2006)].

### Place learning and spatial flexibility depend on the HPC

HPC-lesioned mice failed to acquire the place learning task as well as its relearning, as expected from numerous classic rat studies (McDonald and White, 1994; Packard and McGaugh, 1996; McDonald et al., 2006). It is highly unlikely that this can be better explained by the hyperactive phenotype seen in HPC-lesioned mice as differences were not detectable within the 1st min which is the relevant timeframe for the WCM task. It can be also excluded, that the deficit is due to general motor impairments, because later it is shown that lesioned animals still can learn the response strategy. Actually we could demonstrate that HPC-lesioned mice acquire a compensatory response under place protocol reflected by a high start bias. Interestingly, many HPC-lesioned mice entered the wrong arm, but no longer swam to its end during later stages of training. This resulted in the seemingly contradicting situation of accuracy levels indicating random performance (around 50%), but relatively low wrong-platform values at the same time. Similar behavior of HPC-lesioned rats (enter wrong arm, but turn around well before the end) is seen upon reversal learning in a dry H-maze and was termed “Oops-effect” (Hughey and Koppenaal, 1987). In HPC-lesioned mice it is described that they fail to discriminate correctly amongst two arms in a radial arm maze, if these are presented simultaneously, but can still form go/no-go associations if only one arm at a time is presented (Etchamendy et al., 2003; Mingaud et al., 2007). In our test, once a mouse swam inside an arm, only the cues of this arm are visible, as the opposing ones are behind its back. A HPC-independent go/no-go rule could then take effect and offer an explanation for the seemingly conflicting data.

HPC-lesioned mice could successfully acquire the response protocol per se as well as the free choice protocol. As the lesion of the HPC disrupted place learning, we supposed that lesioned animals undergoing free choice training had to rely on a response-based solution as well. This corroborates findings in rats, where HPC inactivation may even facilitate acquisition of a response (Packard and McGaugh, 1996; McDonald et al., 2002; Stringer et al., 2005). However, in our mice relearning upon free choice training was abolished by HPC-lesions; HPC lesions in rats impaired reversal of a response strategy but did not completely block it (Oliveira et al., 1997). This again speaks in favor of a higher behavioral rigidity of mice compared to rats. We conclude from these data that place learning in mice depends on the hippocampus and is essential for spatial flexibility.

### Residual volume of the HPC reflects relearning capabilities

From the clear bimodality in animals' performance on d5_r_ with some mice showing accurate performance and others failed completely to locate the new platform position correctly (Figure 8B), we assumed that free choice relearning may require a minimum remaining volume of the total HPC. Therefore, we quantified the extent of the HPC lesions for each mouse by means of *in vivo* MEMRI, and set the residual hippocampal volume into relation to individual relearning capabilities. Bilateral injections of the IA reduced total HPC volume by ~50%. On closer inspection it became evident that a remaining volume of 36.9 ± 3.4% was not sufficient for relearning, while 47.7 ± 2.7% was. The responsible cellular process accounting for the threshold is an open question for the future. However, a recent publication from Kassem et al. (2012) suggests, that stress induced gray matter loss in the HPC in mice measured by high-resolution MRI results from a loss of dendrites and their synapses rather than the number or volumes of neuronal somas, astrocytes, or oligodendrocytes. The authors estimate that neurons account for ~66% of the HPC volume. Therefore, neuronal cell loss is a supposable underlying mechanism for the threshold of more than 60% volume loss reported here. The existence of such a threshold is also supported by a study from Moser et al. (1995), where rats with less than 60% intact dorsal HPC showed deficits in place learning, while lesions in the ventral part could not disrupt place learning at all. Here the remaining HPC tissue seems to be unaffected in terms of electrophysiology and cholinergic activity. In our study, the remaining volume of the left HPC and, in particular, the ventral part of the left HPC best reflected the differences in relearning and showed the strongest relationship to the relearning accuracy. The ventral and the dorsal HPC are functionally different structures [for review see Moser and Moser (1998); Fanselow and Dong (2010)]. While the dorsal HPC has a strong role in spatial learning (Pothuizen et al., 2004), the ventral HPC is of importance for emotional behavior such as fear and extinction (Kjelstrup et al., 2002; Sierra-Mercado et al., 2011). Yet, the importance of the ventral HPC for behavioral flexibility has been demonstrated in a number of studies via permanent lesions or transient inactivation (Ellen and Wilson, 1963; Hirsh, 1970; Eichenbaum et al., 1988). In terms of spatial flexibility, rats with lesions of the ventral HPC showed strong perseverance in a short delay spontaneous alternation task in a T-maze as well as impairments in a probability learning task that requires them to change their learned search behavior from one arm (their initially preferred and rewarded one) of the maze to the other (Stevens and Cowey, 1973).

Notably, the remaining volume of the left dorsal HPC correlated with the rearing duration in two paradigms, the open field and the hole board test. Rearing resembles exploration of novel environments (open field) or known environments with novel features (hole board); spatial mapping via the dorsal HPC place cells is thought to contribute to rearing in novel places (Frank et al., 2004; Lever et al., 2006). Moreover, complete HPC lesions in rats prevent the increase in rearing in a novel room (Moses et al., 2002).

Not yet clear is, whether the ventral hippocampus is a direct regulator of the relearning capabilities or rather acts indirectly via stress responses. Keeping in mind (1) the importance of the ventral HPC in negative feedback of corticosterone secretion (Radley and Sawchenko, 2011), and (2) that stress favors response at the expense of place learning strategies (Packard and Wingard, 2004; Elliott and Packard, 2008; Schwabe et al., 2010), it is possible that higher levels of volume reduction of the left ventral HPC results in exaggerated stress responses and, thus, prevention of residual place learning capabilities.

## Conclusion

Taken together, we have validated the WCM as a tool for mice that reliably allows differentiation between navigational strategies from the first day of training on as well as selective training and testing for these strategies. By using the WCM we demonstrated that relearning was impossible in C57BL6/N mice, if only response-based strategies could be employed. Place learning, in contrast, enabled relearning and, therefore, spatial flexibility. With the help of IA-induced lesions we demonstrated the importance of HPC-based strategies for acquiring a new platform position. A volume reduction of the total HPC by more than 60% completely disrupted relearning, whereby volume changes in the ventral part of the left HPC best reflected relearning differences. Hence our data support an important role of the HPC for spatial flexibility in mice.

### Conflict of interest statement

The authors declare that the research was conducted in the absence of any commercial or financial relationships that could be construed as a potential conflict of interest.
